# Structural validity of the Arabic version of the disabilities of the Arm, Shoulder and Hand (DASH) using Rasch measurement model

**DOI:** 10.1186/s41687-021-00392-0

**Published:** 2021-11-06

**Authors:** Ali H. Alnahdi

**Affiliations:** Rehabilitation Sciences Department, College of Applied Medical, Sciences, King Saud University, P.O. Box 10219, Riyadh, 11433 Saudi Arabia

**Keywords:** Upper limb, Validity, Psychometrics, Measurement properties, Outcome measure

## Abstract

**Background:**

The disabilities of the arm, shoulder and hand (DASH) is a commonly used region-specific patient-reported outcome measure (PROM) that quantify upper extremity function (activity limitation) and symptoms. Current evidence suggests that measurement properties of the adapted versions of the DASH are not sufficiently examined. The Arabic DASH has evidence supporting its internal consistency, test–retest reliability, construct validity and responsiveness. On the other hand, the validity of the assumed unidimensionality of the Arabic DASH has not been examined previously. The aim of this study was to examine the structural validity of the Arabic DASH in patients with upper extremity musculoskeletal disorders using Rasch measurement model.

**Methods:**

Patients with upper extremity musculoskeletal disorders were recruited and were asked to complete the Arabic DASH at their initial visit to physical therapy departments. The overall fit of the Arabic DASH to the requirement of the Rasch measurement model was examined using chi-square statistics for item-trait interaction, mean item and person fit residuals. The fit of individual items, thresholds ordering, local dependency, differential item functioning (DIF), and unidimensionality using the t-test approach were also examined.

**Results:**

The Arabic DASH did not fit the Rasch measurement model initially (χ^2^ = 179.04, *p* < 0.001) with major breach of local item independence and a pattern of high residual correlations among the activity-related items and among the impairment-related items. Combining items into activity-limitation and impairment testlets accommodated the local dependency and led to satisfactory fit of the Arabic DASH to the requirement of the Rasch measurement model (χ^2^ = 3.99, *p* = 0.41).

**Conclusions:**

Rasch measurement model supports the structural validity of the Arabic DASH as a unidimensional measure after the accommodation of local dependency.

## Background

The disabilities of the arm, shoulder and hand (DASH) is a commonly used region-specific patient-reported outcome measure (PROM) that quantify upper extremity function (activity limitation) and symptoms [1]. Early evidence supported the reliability, construct validity and responsiveness of the DASH in patients with upper extremity musculoskeletal disorders [2–4]. Given its widespread use, DASH has been cross-culturally adapted to large number of cultures and languages [5, 6]. de Klerk et al. indicated in a systematic review that many adapted versions of the DASH suffer from inadequate testing of measurement properties [7]. The vast majority of the studies in this systematic review did not establish the structural validity of the adapted DASH versions [7].

The measurement properties of the Arabic version of the DASH has only been tested in one single study [8]. Alotaibi et al. completed the cross-cultural adaptation (forward translation then backward translation followed by expert committee review and pilot testing) of the scale into Arabic language then examined the internal consistency, test–retest reliability, measurement error, construct validity and responsiveness of the Arabic DASH [8]. The results of their study supported the measurement properties examined. In this study the authors used the common scoring method of the scale using one summary score for the whole scale which assume that all items are reflections of one underlying construct and that the scale is fairly unidimensional. The validity of the assumed unidimensionality of the Arabic DASH was not tested by the authors.

The consensus-based standards for the selection of health measurement instruments (COSMIN) defines structural validity as “the degree to which the scores of a health-related patient-reported outcomes instrument are an adequate reflection of the dimensionality of the construct to be measured” [9]. Establishing structural validity of multi-item PROM is imperative to ensure that the scale score (either total score or different subscales) actually reflects the constructs measured. The DASH contain items that capture upper extremity activity limitation and also items that capture psychosocial aspects and impairments in body function. These different domains covered by the DASH, although important and relevant to patients with upper extremity disorders [10–12], might threaten the unidimensionality of the measure and question the use of one total score as indicated previously using both classical and modern test theory methods [13–19]. Thus summarizing a scale with potential multiple constructs in one single score would be invalid and greatly limits the interpretation of the scale scores.

In addition to the unestablished dimensionality of the Arabic DASH, no prior studies examined the validity of the Arabic DASH 5 response categories, and whether the scale items function in a similar manner among subgroups of patients with different characteristics (measurement invariance). In contrast to classical test theory methods, Rasch measurement model provide unique opportunity to examine the Arabic DASH structural validity and to examine the validity of the rating scale and also to examine its measurement invariance across important clinical characteristics [20–22]. Therefore, the aim of this study was to examine the structural validity (internal construct validity) of the Arabic DASH in patients with upper extremity musculoskeletal disorders using Rasch measurement model.

## Methods

### Setting and participants

Participants in the current study were recruited from two outpatient physical therapy departments in Riyadh city using convenience sampling. Patients with upper extremity musculoskeletal disorders were recruited if they were 18 years of age or older. Potential participants were excluded if they were unable to read or understand Arabic language, or if they had any neurological disorder. Participants were also excluded if they had spinal surgery or disorder, cardiopulmonary disorder, or neurological disorder that were perceived by the patients as functionally limiting. Ethical approval for the study was obtained from the ethical committees at the participating institutes. Participants signed informed consent forms prior to participation. Two licensed physical therapists and one licensed occupational therapists with cumulative clinical experience of 20 years collected the data.

### Procedure

Participants were asked to complete the Arabic DASH during their initial visit to the physical therapy department where they were seeking care for their upper extremity disorders. The Arabic DASH is 30-item region-specific PROM. Each item was scored on 1 (no functional limitation and no symptoms) to 5 (functional inability and extreme symptoms) scale [8]. Items response categories are “no difficulty”, “mild difficulty”, “moderate difficulty”, “severe difficulty”, “unable” for 21 items, and “none” “mild”, “moderate”, “severe”, “extreme” for 5 items. Each of the remaining four items has different response categories. The typical 0–100 DASH scores can be obtained by subtracting 1 from the mean items score then multiplying by 25. Higher scores in the Arabic DASH indicates greater activity limitation and worse symptoms.

### Statistical analysis

#### Rasch analysis

Partial credit model [23] was used to examine the satisfaction of the Arabic DASH to the requirement of the Rasch measurement model using the RUMM2030 software [24]. The overall fit of the Arabic DASH to the Rasch model was examined using chi-square statistics for item-trait interaction [21, 25]. A non-significant chi-square statistics suggest an overall fit of the measure to the Rasch model and a mean item and person fit residuals close to 0 with standard deviation close to 1 were used as indicators of good fit. Deviation of individual item and person from the Rasch measurement model was examined using standardized fit residuals and chi-square statistics. Standardized fit residuals above or below ± 2.5 and a significant chi-square statistics (after Bonferroni correction) were used to indicate deviation of individual item from the measurement model [21, 25]. The item fit residuals are the sum (across persons) of standardized squared differences between the observed score and the expected score by the model transformed to approximate normal distribution (mean 0, standard deviation of 1) under the hypothesis of good fit to the measurement model [25]. Items were examined for any disordered thresholds by inspecting the category characteristic curves of each individual item [20, 21, 26, 27]. Violation of the local item independence assumption was examined by testing the residual correlation between items [21, 28, 29]. Pairwise residual correlation 0.2 above the mean residual correlation was used to indicate violation of local item independence. The Arabic DASH items were also examined to see whether these items function in the same way in different patients’ subgroups. Differential item functioning (DIF) were examined for sex (male vs. female), age (< 42 years vs. ≥ 42 years; split by median), surgical status (surgery vs. no surgery), and affected side (dominant vs. non-dominant) [21, 27]. Two-way ANOVAs (class interval by subject characteristics) on Rasch residuals were used to detect items uniform and non-uniform DIF. The t-test approach was used to examine the unidimensionality of the Arabic DASH [21, 30]. Principal component analysis on residuals was perform first then the pattern of loading on the first component was used to group items into 2 group (items with positive loadings and items with negative loadings). Each participant’s upper extremity function was then estimated twice using the 2 groups of items then these estimates were compared using t-test. The Arabic DASH was considered unidimensional if the number of significant differences between the 2 estimates was not more than 5%. Person Separation Index was used to examine the internal consistency of the Arabic DASH.

### Sample size estimation

The required sample size was determined based on the COSMIN guidelines [31]. A sample size of 100 participants was considered adequate by the COSMIN guideline for structural validity testing using Rasch analysis [31], thus 100 was used as the minimum required sample in the current study.

## Results

One hundred and nine participants with upper extremity disorders were enrolled (Table 1) (Table 2). Fifty participants (45.9%) had no missing items on the Arabic DASH while 59 participants (54.1%) had 1 missing item (no response). The only item that was missed by the participants was item 21 (sexual activities) which is an optional item in the Arabic DASH and thus was removed before conducting the Rasch analysis.

**Table 1:** Characteristics of participants (N = 109)

Variable	Mean (SD) or *N* (%)
Age (year)	42.1 (14.5)
*Sex*	
Male	63 (57.8)
Female	46 (42.2)
*Height (m)*	1.67 (0.09)
Male	1.72 (0.07)
Female	1.59 (0.06)
*Mass (Kg)*	77.32 (15.43)
Male	81.65 (14.66)
Female	71.39 (14.61)
Body mass index (Kg/m^2^)	27.89 (5.4)
Arabic DASH (0–100)*	42.34 (22.22)
*Site of disorder*	
Shoulder and arm	69 (63.3)
Elbow and forearm	12 (11)
Wrist and hand	28 (25.7)
*Upper extremity surgery*	
Yes	40 (36.7)
Time after surgery (months)	3.73 (7.62)
No	69 (63.3)
Duration of symptoms (months)	7.31 (10.39)
*Affected side*	
Dominant	72 (66.06)
Non-dominant	37 (33.94)

*computed using DASH typical scoring method: (mean items score − 1) × 25

**Table 2:** Type of participants’ musculoskeletal disorder

Shoulder and arm (N = 69)	Elbow and forearm (N = 12)	Wrist and hand (N = 28)
Rotator cuff disorders (25)	Tennis elbow (3)	Fracture (14)
Frozen shoulder (4)	Golfer’s elbow (1)	Dislocation (1)
Shoulder arthroscopy (2)	Arthroscopic debridement (2)	Tendon transfer (1)
Bankart repair (5)	Fracture (3)	Tenolysis (1)
Rotator cuff repair (11)	Dislocation (1)	Trigger finger release (1)
Subacromial decompression (2)	Capsular release (2)	Thumb pain (1)
Osteoarthritis (2)		Wrist pain (1)
Nonspecific shoulder pain (5)		Ligament tear (1)
Fracture (6)		DeQuervain Tenosynovitis (2)
Dislocation (6)		Ganglion cyst (1)
Humeral allograft (1)		Surgical fixation (4)

Initially, Rasch analysis suggested that the Arabic DASH deviates from the Rasch measurement model as indicated by the significant chi-square statistics for item-trait interaction and the high standard deviations for item and person fit residuals (Table 3). The initial analysis identified 18 misfitting patients, 4 misfitting items (items 26 (tingling), 28 (stiffness), 29 (sleep), and 30 (confidence)), 12 items with disordered thresholds (items 1 (open a jar), 2 (write), 3 (turn key), 4 (prepare meal), 8 (garden/yard work), 11 (carry heavy object), 15 (put on sweater), 16 (use a knife), 18 (recreational: Force), 22 (Interference with social activities), 29 (sleep), and 30 (confidence)) and 44 item pairs with high residual correlation indicating local dependency. Four items exhibited uniform DIF by sex (items 1 (open a jar), 4 (prepare meal), 5 (open heavy door), 19 (recreational: Free arm)) while none of the items had uniform or non-uniform DIF by age, surgical status, or affected side. At this stage the t-test approach also did not support he unidimensionality of the Arabic DASH (Table 3).

**Table 3:** Rasch analysis results at each run

Run	Analysis	N	Item fit residual		Person fit residual		Item-trait interaction		PSI	Unidimensionality T-tests
			Mean	SD	Mean	SD	χ^2^ (df)	*P*		% of significant tests
1	Initial analysis	109	0.168	1.824	− 0.152	1.741	179.04 (58)	< 0.001	0.959	19.27%
2	18 misfitting patients removed	91	0.066	1.486	− 0.009	1.169	153.30 (58)	< 0.001	0.962	15.38%
3	Items grouped into two testlets	91	− 0.016	1.964	− 0.463	0.901	3.99 (4)	0.407	0.934	3.30%

SD = standard deviation; χ^2^ = Chi-square; df = degrees of freedom; PSI = Person separation index

**Fig. 1:**
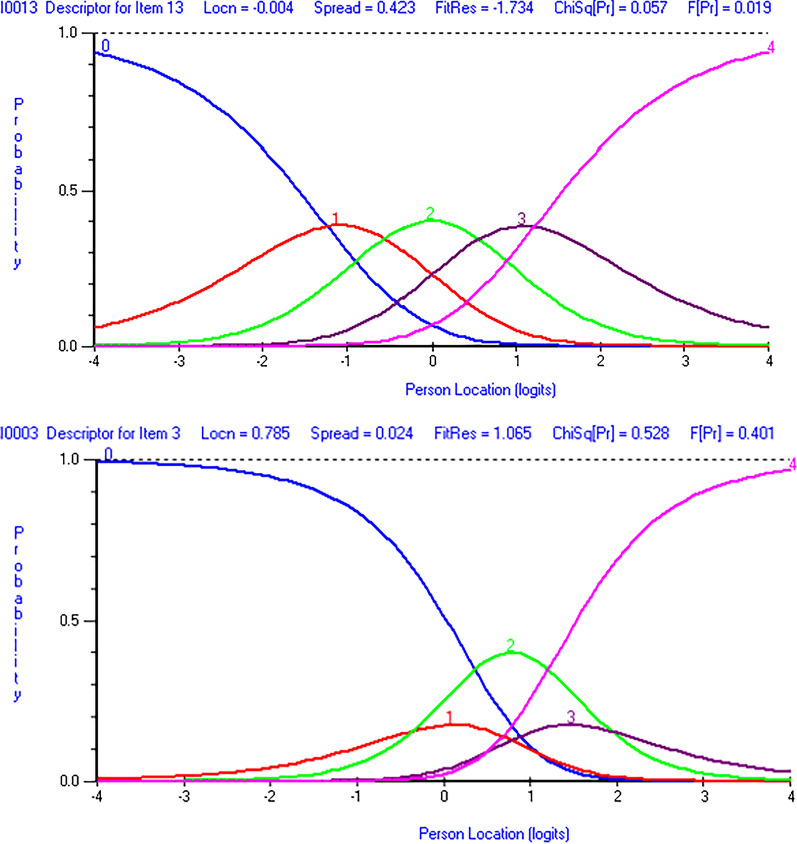
Category characteristic curves after removing misfitting patients (run number 2) and before grouping items into two testlets. Item 13 (wash your hair) in the top figure and item 3 (turn a key) in the bottom figure. The top figure shows properly functioning response categories with ordered threshold. The bottom figure shows dysfunctional response categories with disordered thresholds. Lower person location in the horizontal axis indicate better upper extremity function. 0 = no difficulty, 1 = mild difficulty, 2 = moderate difficulty, 3 = severe difficulty, 4 = unable

After removing the misfitting patients in the second run, the Arabic DASH still did not satisfy the requirement of the Rasch measurement model showing significant chi-square statistics for item-trait interaction and its unidimensionality was not supported (Table 3). At this stage, the scale had 3 misfitting items (items 26 (tingling), 29 (sleep), and 30 (confidence)), 11 items with disordered thresholds (Table 4) (Fig. 1) and 37 item pairs with high residual correlation (Appendix 1). None of the items at the second run exhibited uniform or non-uniform DIF by age, sex, surgical status, or affected side.

**Table 4:** Items Hierarchy and fit statistics (items arranged from most difficult to easiest) after removing misfitting patients (run number 2) and before grouping items into two testlets

Item	Location	SE	Fit residual	χ^2^	P^*^	Ordered thresholds
7 Do heavy household jobs	− 1.39	0.13	− 1.71	4.46	0.108	✓
19 Recreational: Free arm	− 1.09	0.12	0.37	0.58	0.747	✓
11 Carry heavy object	− 1.03	0.12	− 0.36	0.32	0.853	×
25 Pain during activity	− 1.02	0.14	0.34	1.37	0.503	✓
18 Recreational: Force	− 0.89	0.12	0.01	2.55	0.279	×
12 Change bulb overhead	− 0.69	0.12	− 0.63	2.94	0.230	✓
14 Wash your back	− 0.65	0.12	− 0.60	0.50	0.781	✓
6 Place on a shelf	− 0.29	0.13	− 0.75	1.59	0.452	✓
24 Pain	− 0.27	0.14	− 0.82	2.40	0.301	✓
27 Weakness	− 0.24	0.12	0.75	0.13	0.937	✓
1 Open a jar	− 0.21	0.12	1.92	0.97	0.616	×
5 Open heavy door	− 0.18	0.13	− 0.89	5.95	0.051	✓
13 Wash/blow dry hair	0.00	0.12	− 1.73	5.72	0.057	✓
10 Carry shopping bag	0.02	0.12	− 1.32	3.71	0.156	×
8 Garden/yard work	0.03	0.11	− 1.13	1.19	0.550	×
28 Stiffness	0.13	0.11	0.60	7.12	0.029	×
23 Limitation in work and daily activities	0.22	0.13	− 0.67	2.09	0.351	✓
29 Sleep	0.26	0.12	2.14	29.43	< 0.001	×
9 Make bed	0.35	0.12	− 1.50	3.21	0.201	✓
16 Use a knife to cut food	0.47	0.12	− 1.75	4.84	0.089	✓
20 Manage transportation needs	0.54	0.12	0.88	1.76	0.415	✓
4 Prepare meal	0.65	0.12	0.22	3.89	0.143	✓
26 Tingling	0.67	0.12	3.53	29.93	< 0.001	✓
22 Interference with social activities	0.69	0.12	1.38	1.74	0.419	×
30 Confidence	0.72	0.12	4.20	29.04	< 0.001	×
15 Put on sweater	0.72	0.13	− 0.41	0.70	0.705	✓
3 Turn key	0.79	0.12	1.07	1.28	0.528	×
17 Recreational: little effort	0.84	0.13	− 0.63	2.96	0.228	✓
2 Write	0.88	0.12	− 0.59	0.96	0.620	×

Negative item location indicate harder items while positive location indicate easier items

SE = Standard error; χ^2^ = Chi-square.* With Bonferroni adjustment for 29 items (0.05/29), *p* value of less than 0.0017 was considered significant

**Fig. 2:**
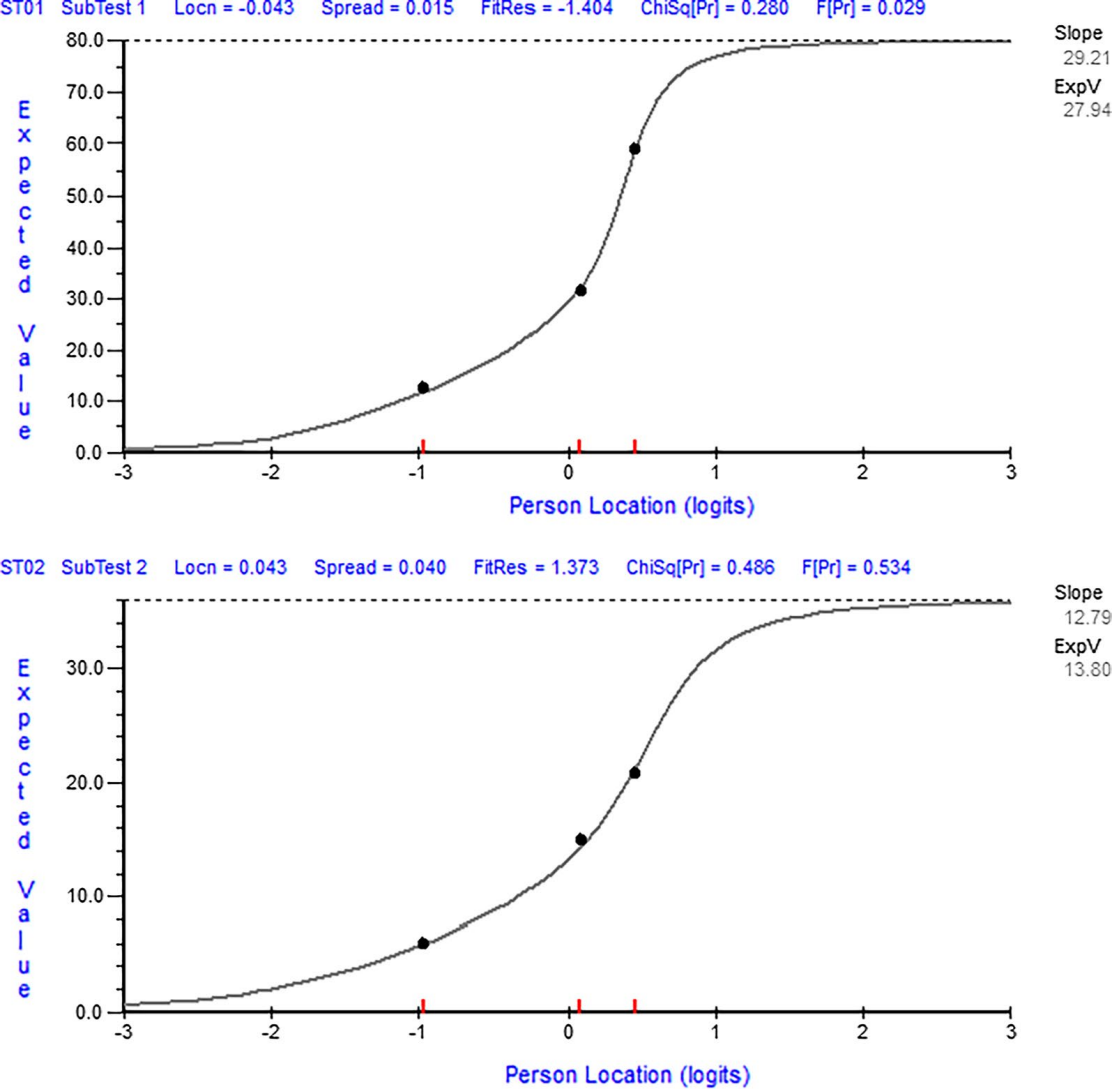
Item characteristic curves for the two testlets (run number 3). Top figure (activity limitation testlet) and bottom figure (impairment testlet) shows fitting items where the observed data (black dots representing data of three groups of subjects with low, moderate, and high upper extremity function) follow the pattern expected by the measurement model (gray line)

**Fig. 3:**
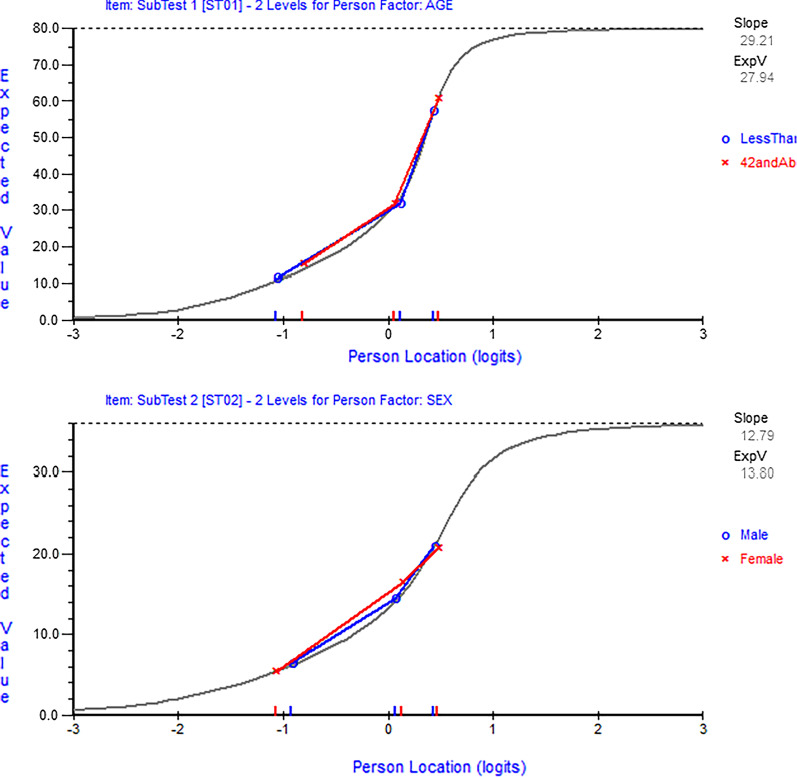
Item characteristic curves for the two testlets (run number 3). Top figure (activity limitation testlet) shows absence of DIF by age and bottom figure (impairment testlet) shows absence of DIF by sex

In the third run of the analysis, items within the Arabic DASH were grouped into 2 testlets to accommodate for local dependency (Table 3). The activity limitation testlet (super item) included activity-related items (items 1 to 20) while the impairment testlet included mostly impairment-related items (items 22 to 30). This grouping of items was determined based on the pattern of residual correlations among items (Appendix 1). At this stage, the Arabic DASH satisfied the expectations of the Rasch measurement model as indicated by the non-significant chi-square statistics for item-trait interaction (Table 3). The 2 super items fitted the Rasch model (activity limitation testlet; fit residual =  − 1.40, χ^2^ = 2.55, *p* = 0.280) (impairment testlet; fit residual = 1.37, χ^2^ = 1.44, *p* = 0.486) (Fig. 2) and had no uniform or non-uniform DIF by age, sex, surgical status, or affected side (Fig. 3). The t-test approach indicated that only 3.3% of the participants showed significant difference between the two ability estimates (using the two testlets) supporting the unidimensionality of the scale (Table 3). Additionally, using the 2 testlets retained 97% of the common non-error variance further supporting the presence of one general factor. The Arabic DASH had good internal consistency with Person Separation Index of 0.93 which decreased from 0.96 after accounting for local dependency. Given the fit of the Arabic DASH to the Rasch measurement model after the creation of 2 testlets, Table 5 allows the transformation of the total raw ordinal-level scores (items scored from 0 to 4 with a total score from 0 to 116 for the 29 items) to an interval-level scores (0–100) with 100 representing worst upper extremity function. The transformation to the interval-level scores would allow the use of parametric statistics and would enhance the validity and interpretation of the scale change scores [32].

**Table 5:** Arabic DASH total raw ordinal-level score (0–116) to interval level score (0–100) with 100 representing worst upper extremity function

Raw score	Interval-level score	Raw score	Interval-level score	Raw score	Interval-level score	Raw score	Interval-level score
0	0.0	30	54.6	60	66.9	90	71.6
1	10.0	31	55.3	61	67.1	91	71.8
2	16.2	32	56.0	62	67.2	92	72.1
3	20.1	33	56.7	63	67.4	93	72.3
4	23.0	34	57.4	64	67.5	94	72.5
5	25.3	35	58.0	65	67.7	95	72.8
6	27.3	36	58.6	66	67.8	96	73.1
7	29.1	37	59.1	67	68.0	97	73.4
8	30.7	38	59.7	68	68.1	98	73.7
9	32.2	39	60.2	69	68.3	99	74.0
10	33.7	40	60.7	70	68.4	100	74.4
11	35.1	41	61.2	71	68.5	101	74.7
12	36.4	42	61.6	72	68.7	102	75.1
13	37.7	43	62.1	73	68.8	103	75.6
14	39.0	44	62.5	74	69.0	104	76.0
15	40.2	45	62.9	75	69.1	105	76.5
16	41.4	46	63.3	76	69.3	106	77.1
17	42.5	47	63.6	77	69.4	107	77.7
18	43.7	48	64.0	78	69.5	108	78.4
19	44.8	49	64.3	79	69.7	109	79.2
20	45.8	50	64.6	80	69.8	110	80.1
21	46.8	51	64.9	81	70.0	111	81.3
22	47.8	52	65.1	82	70.2	112	82.7
23	48.8	53	65.4	83	70.3	113	84.5
24	49.7	54	65.7	84	70.5	114	87.3
25	50.6	55	65.9	85	70.6	115	91.9
26	51.5	56	66.1	86	70.8	116	100.0
27	52.3	57	66.3	87	71.0		
28	53.1	58	66.5	88	71.2		
29	53.9	59	66.7	89	71.4		

For the raw ordinal-level scores, items scored from 0 to 4 with a total raw score ranging from 0 to 116 for the 29 items

## Discussion

The Arabic DASH was subjected to the requirements of the Rasch measurement model in the current study. Initially, the Arabic DASH did not satisfy the requirements of the Rasch measurement model with several areas of deviations including misfitting items, dysfunctional response categories, significant violation of local item independence, and lack of unidimensionality. After the accommodation of the local dependency by creating 2 super items reflecting upper extremity activity limitation and impairment, the Arabic DASH satisfied the requirement of the Rasch measurement model indicating that the scale if fairly unidimensional measure of upper extremity activity limitation and impairment.

Item 21 (sexual activities) was the only item that had missing responses (no response). In the Arabic DASH, item 21 (sexual activities) was the only item that was labeled as an optional item which might explain why only this item had missing responses. Given that item 21 cover sensitive issue (sexual activities), cultural factors might have also contributed to some missing responses to this item. The optional nature of the item and the high missing rate led to the removal of the item 21 (sexual activities) before conducting Rasch analysis in the current study.

After removing misfitting patients, Items 30 (confidence), 26 (tingling), and 29 (sleep) were misfitting based on their fit residuals and chi-square statistics. This indicate that the behavior of these items did not follow the expectation of the Rasch measurement model. The non-conformity of these items to the Rasch measurement model suggests that these items do not belong to the major underlying trait measured by the majority of the scale items, upper extremity activity limitation. The violation of local item independence observed in the current study might have driven these items to deviate from the measurement model [29, 33]. Although these items were individually misfitting, the activity limitation and impairment testlets that were created to accommodate the local dependency showed good fit to the Rasch measurement model allowing the retention of these items. In patients with rheumatoid arthritis, Prodinger et al. reported good fit of the same 2 testlets (activity limitation and impairment testlets) used in our study providing support to the findings of the current study [34]. Studies that examined the fit of the DASH to the Rasch measurement model reported a number of misfitting items ranging from 1 to 16 items [15–19, 35–40]. All of the misfitting items in our study have been reported in the literature to deviate from the Rasch measurement model in different upper extremity musculoskeletal populations including patients with elbow disorders, shoulder disorders, hand disorders, Dupuytren’s contracture, humeral shaft fracture, and in patients with various upper extremity disorders [15–19, 35–37]. Item 26 (tingling) was the most consistently reported misfitting item across these studies followed by item 30 (confidence). Items 26 (tingling), and 30 (confidence) has also been reported to misfit the Rasch model in patients neurological disorders affecting upper extremity function [38, 39].

Proper functioning of the scale response categories manifest as ordered coverage of the underlying continuum by the response categories where each response category becomes the most probable option in part of the continuum (Fig. 1). Under this ordered coverage, the response option “no difficulty” should be the most probable option for individuals with high level of upper extremity function followed by “mild difficulty”, “moderate difficulty”, “severe difficulty”, then the response option “unable” with decreasing levels of upper extremity function. Improper functioning of the response categories and disordered thresholds were detected in 11 items in the Arabic DASH (Table 4). This indicates that response categories in these items were not used in the expected manner (either because wordings of response categories were not clear, or patients were not able to discriminate between them). Another potential reason for the disordered thresholds in the Arabic DASH is the significant violation of local item independence which could cause items’ thresholds to be disordered [34]. All items with disordered thresholds in the Arabic DASH showed violation of local item independence as indicated by the high residual correlations. Prior studies that examined the internal structure of the DASH also pointed to disordered thresholds suggesting improper functioning of the scale response categories [15–18, 35, 37–39]. The number of items with disordered thresholds reported in the literature ranged from 2 items in patients with hand and elbow disorders [15, 35] to 19 items in patients with various upper extremity disorders [18].

The Arabic DASH has major violation of local item independence indicating response dependency and multidimensionality [28, 33]. The Arabic DASH suffered from local dependency between 37 item pairs. This indicates that these items have something in common other than the measured underlying trait that is upper extremity function violating the requirement of local item independence [20, 21, 27, 29]. This violation caused the scale to deviate from the Rasch measurement model. The accommodation of local dependency within the scale by creating testlets (activity limitation and impairment testlets) led to satisfactory fit of the Arabic DASH to the Rasch measurement model supporting the validity of the scale. Examining the item pairs with residual correlations above the predetermined threshold indicates that most of these items inquire about similar functional activities or symptoms. Items 18 (recreational: force) and 19 (recreational: free arm) had the highest bivariate residual correlation after the removal of the underlying trait “upper extremity function”. Both items inquire about the difficulty encountered in recreational activities while taking some force through the arm (item 18) and in free arm movement (item 19). The similar content of the 2 items and possible redundancy might explain the high residual correlation observed. Items with the second highest residual correlation were items 10 (carry shopping bag) and 11(carry heavy object). Both items are related to the same functional activity that is “carrying” and response to one item is likely to be linked or dependent on the response of the other item. For example if a patient was able to carry a heavy bag over 4.5 kg (item 11) then that patient would be able to carry a shopping bag (item 10). This problem of response dependency detected in the Arabic DASH is of concern given that it artificially inflates reliability and influence person estimates [28, 29].

Similar to the residual correlation observed between activity-related items, impairment-related items also exhibited high residual correlation. This pattern of residual correlation might be an indicator of multidimensionality where these items represent an impairment-related dimension. Additionally, similar content might also explain some of the residual correlations among impairment-related items. Items 24 (pain), 25 (pain during activity), and 29 (sleep) for example inquire about pain severity and pain-related sleep difficulty. This enquiry about the same impairment might constitute the shared concept that lead to the high residual correlation even after the removal of the major underlying factor. Consistent with our findings, DASH has been reported to violate the requirement of local item independence. A pattern of high residual correlation similar to ours where activity-related item group together while impairment-related items group together has been reported in patients with various upper extremity musculoskeletal disorders [15, 16, 18, 34, 36, 37]. Similar to the approach used in the current study, Prodinger et al. reported the use of two testlets (activity limitation and impairment testlets) to accommodate the issue of local dependency within the scale and this method yielded satisfactory fit to the Rasch model in line with the findings of the current study [34].

Rasch measurement model is a unidimensional model where the probability of being able to perform an activity is only governed by a single factor that is the person ability (level of upper extremity function possessed by the patient). We believe that the major breach of local item independence was the main reason explaining why the Arabic DASH did not satisfy the requirement of unidimensionality initially. After the accommodation of the local dependency by the creation of activity limitation and impairment testlets, The Arabic DASH satisfied the requirement of unidimensionality as suggested by the principal component analysis of residuals followed by the t-test [29, 33]. Although items were grouped into two groups similar to having two subscales, the majority of the common non-error variance was retained by the two testlets suggesting that the scale has one general factor [34]. These results support the unidimensionality of the Arabic DASH and supports the validity of providing one single summary score for the Arabic DASH. Similar to the findings of the current study, Prodinger et al. reported that the DASH was sufficiently unidimensional in patients with rheumatoid arthritis after addressing the issue of local dependency between items using testlets [34]. The accommodation of local dependency through the use of testlets also led to unidimensionality of the Finnish DASH in patients with hand and wrist disorders[15]. Lack of unidimensionality of the DASH was reported in the literature in patients with hand disorders [16], Dupuyteren’s contracture [17], various upper extremity musculoskeletal disorders [18, 19], shoulder disorders [36] and also in patient with stroke [40]. Number of these studies did not examine for violation local item independence [17, 19, 40]. On the other hand, the studies that examined for this violation used high threshold for detecting local dependency thus underestimated the degree of dependency and also did not examine the effect of the accommodation of dependency using testlets on scale unidimensionality [16, 18, 36].

The Arabic DASH items seems to have no uniform or non-uniform DIF by age, sex, surgical status, or affected side. This suggests that the scale items behave in a similar manner regardless of the patient characteristics and that items were not biased to any of the levels of these characteristics (for example bias toward males versus females). The lack of uniform and non-uniform DIF was also observed in the current study at the level of testlets suggesting that the testlets also are invariant to patients’ characteristics. These measurement invariance results of the Arabic DASH at the item level and at the testlet level should be interpreted with caution giving the limited number of participants in each subgroup [31] and a follow-up study might be need to confirm our findings. To the best of our knowledge, the current study is the first study which suggested that the DASH is invariant to the treatment received (surgically or non-surgical) and whether the affected side was the dominant or the non-dominant side. Similar to the findings of the current study, the activity limitation and impairment testlets were reported to have no DIF by age in patients with rheumatoid arthritis and the reported DIF by sex in the testlets was considered trivial and required no modifications [34]. DASH individual items has also been reported previously to have no DIF by sex [36, 37] and age [16]. On the contrary, number of studies reported DIF by sex [15–17, 35, 41] and age [15, 36, 37, 41] in the DASH items but the results of these studies were inconsistent regarding the number of items, and the specific items exhibiting DIF.

This study represents the first attempt to examine the structural validity (internal construct validity) of the Arabic DASH. Rasch measurement model pointed to areas of dysfunction in the behavior of the Arabic DASH items mainly local dependency between items that could not have been determined using classical test theory methods. The accommodation of the local dependency between items improved the internal structure of the Arabic DASH resulting in an interval-level unidimensional measure of upper extremity function. The results of this study would help in guiding future modifications of the scale aiming to improve its validity. Although the sample size used in the current is adequate for conducting Rasch analysis [31], it is at the lower end of what is considered adequate sample size thus further testing of the measure might be needed using larger number of participants to confirm the findings of the current study. Additionally, when the whole sample was split into subsamples for DIF analysis (e.g. male and female), the number of participants within each group was below the recommended number for examining scale invariance [31]. Thus a caution should be practiced when interpreting the findings of the DIF analysis reported in the current study. The majority of participants in the current study have shoulder and arm disorders then wrist and hand disorders with few participants who had elbow and forearm disorders, thus the results of the current should be interpreted with caution especially for patients with elbow and forearm disorders.

## Conclusions

Rasch measurement model supports the validity of the Arabic DASH as a unidimensional measure of upper extremity activity limitation and impairment after accommodating for local dependency between items. The total Arabic DASH score can be used in clinical practice and for research purposes to reflect the level of upper extremity activity limitation and impairment in patients with upper extremity musculoskeletal disorders.

## Data Availability

The datasets used and/or analyzed during the current study are available from the corresponding author on reasonable request.

## Appendix 1

See Table 6.

**Table 6:** Items residual correlations after removing misfitting patients (run number 2) and before grouping items into two testlets

Item	1	2	3	4	5	6	7	8	9	10	11	12	13	14	15
1															
2	*0.178*														
3	0.163	*0.343*													
4	0.127	*0.173*	*0.222*												
5	0.09	0.107	*0.333*	0.116											
6	− 0.068	0.027	− 0.164	− 0.126	0.07										
7	*0.17*	0.025	− 0.047	− 0.014	0.103	0.145									
8	0.109	− 0.005	0.006	− 0.136	0.095	*0.196*	*0.328*								
9	− 0.066	− 0.13	− 0.062	− 0.068	− 0.045	0.091	0.162	*0.389*							
10	− 0.015	− 0.041	− 0.219	*0.248*	0.155	0.016	0.04	− 0.072	0.023						
11	*0.17*	0.018	− 0.194	0.134	0.073	− 0.038	*0.244*	0.008	− 0.026	*0.523*					
12	− 0.06	− 0.082	− 0.14	− 0.078	− 0.118	*0.277*	0.112	0.047	− 0.004	− 0.199	0.142				
13	− 0.2	0.068	− 0.019	0.041	− 0.066	*0.189*	0.109	− 0.116	0.081	− 0.038	− 0.022	*0.232*			
14	− 0.212	− 0.171	− 0.072	− 0.233	− 0.085	*0.207*	0.008	− 0.164	0.011	− 0.274	− 0.203	0.123	*0.325*		
15	− 0.291	− 0.24	− 0.109	− 0.153	− 0.131	0.035	− 0.122	− 0.064	*0.199*	− 0.07	− 0.223	0.087	0.059	*0.198*	
16	0.038	− 0.006	0.166	*0.222*	*0.185*	0.016	− 0.086	0.026	*0.25*	0.005	− 0.006	− 0.07	0.042	0.015	− 0.053
17	− 0.142	− 0.185	− 0.047	0.091	0.012	0.018	− 0.108	− 0.124	*0.175*	0.108	− 0.086	0.017	0.096	− 0.084	− 0.043
18	− 0.174	− 0.11	− 0.118	− 0.129	− 0.074	0.095	0.161	− 0.008	0.089	− 0.013	− 0.046	0.034	*0.204*	− 0.132	− 0.003
19	− 0.108	− 0.23	− 0.103	− 0.266	− 0.249	− 0.029	0.166	− 0.012	0.106	− 0.183	− 0.056	0.117	0.084	− 0.095	0.051
20	− 0.212	− 0.042	0.013	− 0.003	− 0.069	− 0.247	− 0.196	− 0.165	0.011	0.013	0.001	− 0.128	− 0.071	− 0.021	− 0.054
22	0.012	− 0.12	0.149	− 0.111	0.119	− 0.371	− 0.168	− 0.157	− 0.265	− 0.053	− 0.114	− 0.264	− 0.167	0.103	0.063
23	− 0.04	− 0.035	0.146	− 0.059	0.104	− 0.115	− 0.193	− 0.181	− 0.16	0.102	− 0.091	− 0.186	− 0.072	0.052	0.069
24	− 0.103	− 0.114	− 0.11	*0.249*	0.063	0.066	− 0.211	0.046	0.001	0.032	− 0.125	− 0.119	− 0.163	− 0.121	0.163
25	− 0.137	− 0.19	− 0.186	− 0.088	− 0.149	− 0.043	− 0.211	− 0.075	− 0.113	0.051	− 0.06	− 0.092	− 0.273	− 0.135	− 0.056
26	− 0.129	− 0.015	− 0.126	− 0.035	− 0.13	− 0.173	− 0.337	− 0.187	− 0.222	0.002	− 0.152	− 0.133	− 0.246	− 0.078	− 0.123
27	− 0.052	0.012	− 0.023	− 0.129	− 0.125	− 0.212	− 0.131	− 0.129	− 0.036	− 0.076	− 0.046	− 0.034	0.144	− 0.103	− 0.002
28	− 0.036	0.064	0.125	− 0.175	− 0.008	− 0.089	− 0.07	− 0.125	− 0.33	− 0.226	− 0.176	− 0.074	− 0.214	0.065	− 0.103
29	− 0.138	− 0.166	− 0.257	− 0.158	− 0.219	0.058	− 0.273	− 0.044	− 0.197	− 0.111	− 0.109	− 0.194	− 0.281	0.03	0.102
30	*0.269*	− 0.07	− 0.146	− 0.066	− 0.361	− 0.287	− 0.067	− 0.196	− 0.14	− 0.114	− 0.187	− 0.168	− 0.108	0.036	− 0.005

Item	16	17	18	19	20	22	23	24	25	26	27	28	29
1													
2													
3													
4													
5													
6													
7													
8													
9													
10													
11													
12													
13													
14													
15													
16													
17	*0.362*												
18	− 0.064	0.092											
19	− 0.068	0.115	*0.683*										
20	− 0.025	*0.223*	− 0.003	− 0.001									
22	− 0.202	− 0.051	− 0.254	− 0.226	0.077								
23	− 0.15	0.067	− 0.186	− 0.187	− 0.009	*0.517*							
24	*0.203*	− 0.031	− 0.28	− 0.327	− 0.091	− 0.081	0.035						
25	− 0.032	− 0.096	− 0.07	− 0.109	0.092	0.099	0.029	*0.398*					
26	− 0.188	− 0.167	− 0.096	− 0.059	− 0.006	− 0.037	− 0.107	− 0.005	0.099				
27	− 0.096	− 0.272	0.014	0.078	− 0.091	0.027	− 0.051	− 0.014	0.045	0.029			
28	− 0.033	− 0.103	− 0.201	− 0.076	− 0.069	0.059	0.022	− 0.076	0.051	0.134	*0.206*		
29	− 0.24	− 0.167	− 0.149	− 0.062	− 0.053	0.108	− 0.042	*0.251*	0.119	*0.321*	− 0.167	− 0.044	
30	− 0.264	− 0.098	− 0.103	0.012	− 0.033	0.106	− 0.04	− 0.266	− 0.072	*0.287*	0.022	0.002	0.133

Residual correlations above the predetermined threshold (0.2 above the mean residual correlation) are italicized. 1 Open a jar, 2 Write, 3 Turn key, 4 Prepare meal, 5 Open heavy door, 6 Place on a shelf, 7 Do heavy household jobs, 8 Garden/yard work, 9 Make bed, 10 Carry shopping bag, 11 Carry heavy object, 12 Change bulb overhead, 13 Wash/blow dry hair, 14 Wash your back, 15 Put on sweater, 16 Tingling, 17 Recreational: little effort, 18 Recreational: Force, 19 Recreational: Free arm, 20 Manage transportation needs, 22 Interference with social activities, 23 Limitation in work and daily activities, 24 Pain, 25 Pain during activity, 26 Tingling, 27 Weakness, 28 Stiffness, 29 Sleep, 30 Confidence

## Abbreviations

DASH: Disabilities of the arm, shoulder and hand
DIF: Differential item functioning
PROM: Patient-reported outcome measure
COSMIN: Consensus-based standards for the selection of health measurement instruments
ANOVA: Analysis of variance
